# On the Characterisation of the Time-of-Flight VL53L5CX Sensor by STMicroelectronics for Indoor Robotics Applications

**DOI:** 10.3390/s26051639

**Published:** 2026-03-05

**Authors:** Giammarco Caroleo, Alessandro Albini, Perla Maiolino

**Affiliations:** Department of Engineering Science, University of Oxford, Oxford OX1 2JD, UK

**Keywords:** time-of-flight, point clouds, proximity sensing, robotics

## Abstract

Miniaturised proximity Time-of-Flight (ToF) sensors are attractive for robotics applications due to their low cost, compact size, and low power consumption, which makes them suitable for direct distribution on the robot body. However, both the accuracy and the reliability of their measurements are influenced by operating conditions and target properties. These aspects are not fully investigated in the manufacturer’s datasheet, yet they play a crucial role in downstream robotic tasks. To address this gap, we mounted three VL53L5CX sensors, an Ambient Light Sensor, and a thermistor on a robotic manipulator in a controlled laboratory setup and executed a series of experiments to characterise sensor performance. Specifically, experiments were conducted to quantify sensor drift over time, the influence of ambient illumination under three office lighting conditions, within-frame beam variability, depth accuracy over the 20–800 mm range for different materials, orientation sensitivity at different distances, and an empirical signal-to-noise ratio. The results reveal a transient warm-up effect at startup, after which measurements stabilise, a near-linear range-dependent bias with substantially larger uncertainty for dark targets, limited within-frame variability, and an invalid measurement rate consistently below 10%. Overall, the VL53L5CX provides repeatable measurements, and the findings of this work can be leveraged to derive more faithful sensor models, apply range bias correction, and broaden the range of robotic applications.

## 1. Introduction

Miniaturised Time-of-Flight (ToF) sensors have received great attention in a variety of fields ranging from consumable electronics [1,2] and human-pose estimation [3] to material classification and depth imaging [4]. Notably, their compact form factor, low cost and energy efficiency have made them suitable for adoption in robotics applications as well [5,6,7,8]. In fact, ToFs offer advantages over other widespread sensors in robotics, such as RGB-D cameras or LiDARs, in terms of ease of integration, cost, and bandwidth requirements. This is supported by the fact that researchers have successfully used miniaturised ToF sensors for manipulation [5,9,10], navigation [11,12,13] and Human–Robot Interaction (HRI) tasks [14,15]. In [5,9], the authors show how proximity sensing can improve sequential manipulation and grasping tasks respectively, while Lancaster et al. in [10] achieved in-hand object state estimation using distributed ToFs on a robotic gripper. Single-beam proximity sensors are deployed on micro drones in [11,12], enabling Simultaneous Localisation and Mapping (SLAM) operations, while Eshaghi et al. equip millirobots with them to achieve obstacle avoidance in [13]. Similar sensors are used in [14] to perceive human presence and in [15,16] to avoid obstacles in HRI tasks. These works mainly rely on single-beam proximity sensors, which convey limited information, thus hindering the application in more complex scenarios involving cluttered and dynamic environments and more intricate interactions within them.

Recent advancements have enabled ToFs to retrieve sparse distance (multizone) measurements at each frame, rather than just one, which has further expanded their applicability range. Several works have demonstrated the potential of using multizone range sensors to enhance vision [17,18], scene estimation [3,19], obstacle avoidance [6,8,20,21], localisation [22], and perception [7]. In particular, in [17,18], the authors combine a multizone miniaturised ToF with a monocular camera to enhance depth estimation and accomplish SLAM tasks respectively. Human poses are predicted in [3] while Sander et al. exploit distributed multirange proximity sensing to estimate the scene flow in the robot’s surroundings [19]. In [6,21], the same sensors are mounted, respectively, on a lightweight drone and on a miniature mobile robot, to achieve robust and efficient obstacle avoidance. Similarly, in [8,20], the sensors are distributed on robotic manipulators to avoid moving obstacles and accomplish tasks in clutter, respectively. Additionally, in [22], the authors use these sensors for soft robot self-localisation. Giovinazzo et al., instead, propose a combination of proximity and tactile sensors for a novel artificial skin for robots in [7]. In most of these recent works, namely [3,6,8,17,18,19,21,22], the adopted multirange sensor is the VL53L5CX ToF by STMicroelectronics (Geneva, Switzerland) [23]. Despite its advantages, the sensor yields noisy measurements that are affected by several factors such as the distance from the targets, the material properties and the appearance of the targets and the environmental conditions as partially detailed in the datasheet [24]. The effect of such factors is well-known for other range sensors such as LiDARs and radars, as it has been observed and largely investigated in the literature [25,26,27,28,29,30]; comparative analyses proposed in [31,32,33] further suggest that measurement accuracy is affected in various ways for different sensors, and thus a specific characterisation is needed for each of them.

To date, limited investigation has been conducted on the VL53L5CX sensor. The sensor datasheet provides a characterisation but lacks details on sensor drift, as well as assessing how the measurement accuracy depends on the mutual orientation between the target and the sensor, as is commonly analysed for similar sensors [26,34,35]. Further investigation is needed to better characterise the effects of object distance and ambient light as well. Indeed, the measurement conditions described in the datasheet do not specify how many samples are considered and how finely the range has been discretised to retrieve the characterisation curve. Specifically, the datasheet provides uncertainty as a fixed percentage of the measured distance without offset (bias) for a large part of the working range, that is 201–4000 mm, in two ambient light conditions and with both a grey and white target. In the short range, namely between 20 mm and 200 mm, the accuracy is given within a range of ±15 mm, thus oscillating between 75% and 7.5%. To overcome some of these limitations, in [6,36], the authors propose a sensor characterisation for range accuracy considering both white and black targets and two light conditions—ambient indoor light and darkness. Their characterisation spans the 200–3000 mm range in steps of 200 mm. Conversely, Caroleo et al. propose in [37] a preliminary characterisation in which accuracy is investigated with respect to distance from the target and mutual orientation. The range analysed is within 20–800 mm, while the mutual orientation effect is considered with the sensor and the target being within $\pm 20^{\circ }$. However, the analyses are conducted using a white target in ambient indoor light conditions. Accordingly, there are still aspects that need to be characterised.

**Figure 1:**
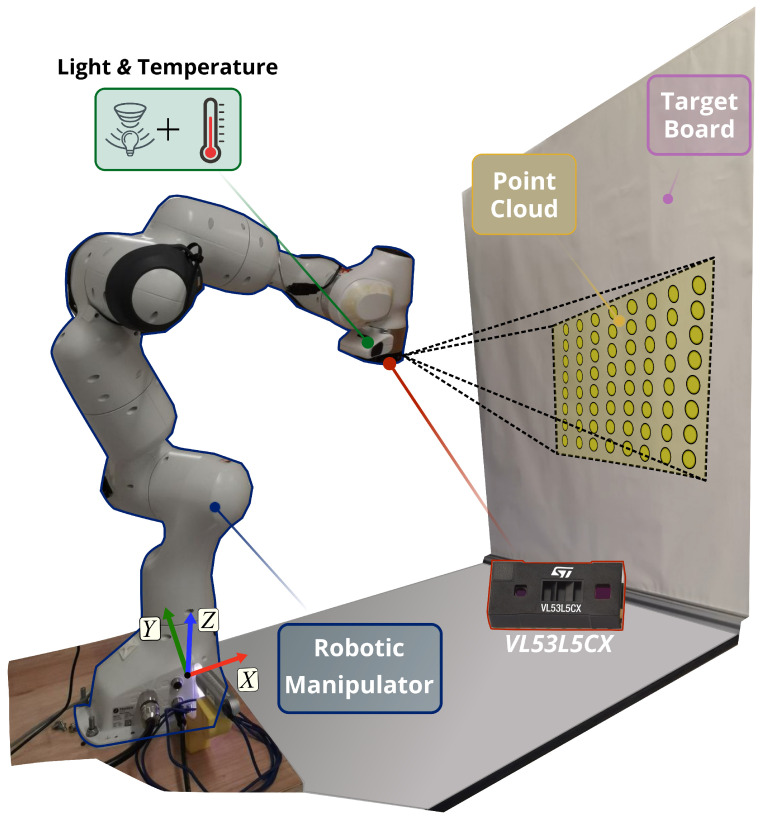
The experimental platform. The miniaturised time-of-flight sensors, namely the VL53L5CX, are mounted on a robotic manipulator that is commanded into different poses with respect to a target board to acquire range measurements in the form of 8 × 8 point clouds. The ambient light and the sensor’s temperature are monitored with relative sensing units during the data collection. In the image, the target board is covered with a white vinyl film.

We argue that it is valuable to conduct a more comprehensive analysis on the VL53L5CX performance in indoor scenarios for robotics applications, given the growing interest shown for the sensor in the recent literature. In particular, in this work, we aim to extend the preliminary work proposed in [37] and characterise the sensor drift, ambient light-induced variations, and the uncertainty on the measurements in the short and medium range, i.e., meaningful distances for robotic manipulators with limited workspace and avoidance of close obstacles. To this end, we propose a systematic approach in which we equip a robotic manipulator with VL53L5CX ToF sensors and command it to move in known positions with respect to a target board. Figure 1 provides an overview of the setup adopted for this manuscript. The multiple beams measurements are collected in the form of point clouds in diverse experimental conditions—obtained by changing the colour of the target board and by actively controlling the ambient light of the environment. The measurements collected allow us to get the information needed to assess sensor performance in different scenarios.

The manuscript is structured as follows. Section 2 presents the methodology and the experimental setup adopted for the characterisation and details how data are collected. In Section 3, we include plots and graphs highlighting sensor performance. In Section 4, key findings are discussed, and insights on practical implications are provided. They follow the conclusions in Section 5.

## 2. Materials and Methods

In this section, we first briefly describe the sensor under investigation and the analyses we conducted. Second, we provide details on the setup used to collect data and highlight the experimental procedures.

### 2.1. VL53L5CX by STMicroelectronics

The VL53L5CX sensor by STMicroelectronics is a multizone ranging sensor which integrates the Single Photon Avalanche Diode (SPAD) array technology, emits in the near-infrared spectrum, i.e., 940 nm, and can measure up to 4 m with a 16-bit resolution. Range measurements come either in 4×4 or 8×8 separate zones and are detected within a squared Field-of-View (FoV) with a $65^{\circ }$ diagonal. When the 4×4 multizone ranging is adopted, the frame rate is 60 Hz while it drops to 15 Hz in the latter case. The sensor weighs 2 g and its dimensions are 6.4×3.0×1.5 mm.

The multizone ranging output provides the absolute distance of an object from the sensor. In order to obtain the raw range measurements, a patented processing technique is used: at each frame, the sensor processes multiple light beams, computes the time it takes for them to be reflected, and bins these values into histograms. This process outputs the most likely value and a flag accounting for the dispersion among the measurements—the range statusthat informs the end user of whether a measurement is reliable or not [24]. However, even measurements flagged as reliable are noisy because of several factors, such as the distance from the target, the orientation with respect to it, and the physical properties of the target and ambient light, to name a few. The effect of these aspects is analysed in Section 3 and discussed in Section 4.

**Figure 2:**
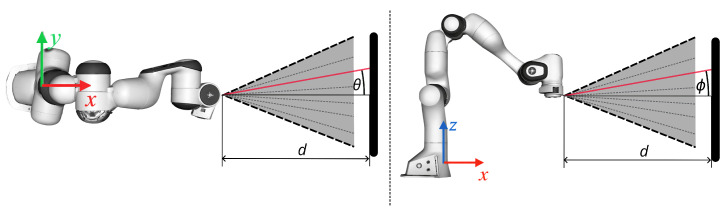
Absolute depth measurement conversion into Cartesian coordinates. The VL53L5CX mounted on the robotic arm measures absolute distance of a target, herein represented by a black board at a distance *d*, and yields 64 measurements. The top and side views show the VL53L5CX FoV in the *x*-*y* and *x*-*z* planes. Beams are equally spaced in a pyramidal FoV, so having θ and ϕ identifying them, it is possible to compute the components of each depth measurement with respect to the axes shown in the figure, hence associating a point in the Cartesian space.

Given the FoV of the emitter, raw distance measurements, $d_{i}$, with i=1,…,64, can be converted into Cartesian coordinates with respect to the sensor origin, producing a point cloud as illustrated in Figure 2, by considering tanθ and tanϕ. The point cloud data structure represents a scalar set $P=\{x_{i}\}$ with $x_{i}\in {ℜ}^{3}$ and is advantageously used in robotics applications spanning from control tasks to obtaining information about the environment [5,8,16,19,38]. Accordingly, for this work, we adopted this convenient structure, which also has the advantage of requiring little memory storage, i.e., below 10 kB, given that at most 64 points are retrieved every frame.

### 2.2. Experimental Setup

**Figure 3:**
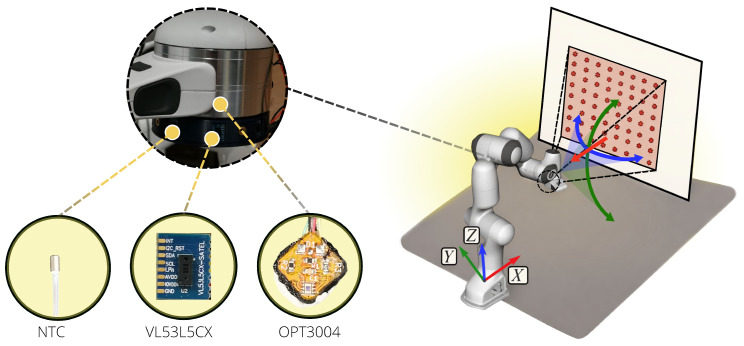
The experimental setup used for the characterisation and the commanded motions for the robot are shown. On the left-hand side of the figure, it can be seen that the robot is equipped with a VL53L5CX sensor measuring the distance, an Negative Temperature Coefficient (NTC) thermistor in contact with another working VL53L5CX not used for data collection, and the PCB with the OPT3004 Ambient Light Sensor (ALS) placed above the VL53L5CX used for data collection so as to be exposed to the same incident light. On the right-hand side, the commanded motions for the robot are shown to characterise the range and mutual orientation influence. The robot is commanded to move along the *x*-axis to characterise the influence of range, as represented with the red arrow, and to move along a segment of a circle both in the *x*-*y* and the *x*-*z* planes to characterise the uncertainty induced on range measurements by orientation as described with the blue and green arrows and shaded areas.

The setup used for the data collection is shown in Figure 3. We mounted three VL53L5CX sensors on a 3D-printed shell, which was attached to the flange of a 7 degrees of freedom Panda Robot by Franka Robotics [39]. The sensors were distributed on a circle at intervals of $45^{\circ }$, occupied known positions in the shell provided by the CAD model, and were connected to a Cypress (Infineon Technologies, Neubiberg, Germany) CY8CKIT-059 PSoC 5LP board for communication. The robotic arm allowed for the estimation of the position of the sensorised shell with respect to its base frame with a precision of the order of $10^{-1}$ mm.

We placed a flat 800×1000 mm board in front of the robot to function as the only detectable obstacle for the sensor. Its position was obtained by measuring distance and orientation with respect to the robot base with a tape measure and a protractor, respectively, with a precision of $10^{-1}$ mm and $10^{-1\circ }$. The board was wrapped on one side with a white vinyl film and covered with 5 mm thick black foam on the other for most of the experiments. This choice was made to represent two reflectivity and colour extremes, bounding expected performance relative to target properties. We further assessed accuracy with the sensor pointing at different materials either by changing the colour of the board surface, e.g., by placing a cloth on it or wrapping it with films and laminates, or by swapping it with another wooden board of similar size but without any wrapping on it.

To monitor the temperature increase on the sensor board and get information on the induced sensor drift, we placed an NTC thermistor on top of one of the sensors using thermal paste. The chosen VL53L5CX was not the one pointing directly at the board to avoid occlusions on the measurements. The resistance measured by the NTC can be converted into a temperature value by using the Steinhart–Hart equation [40]. In particular, the following equation was adopted:

(1) $$\frac{1}{T}=A+B\ln R+C{\left(\ln R\right)}^{3},$$

where *T* is the temperature in Kelvin, *R* is the resistance in Ohms and A,B and *C* are the Steinhart–Hart coefficients that can be computed having three operating points available in the datasheet of the thermistor. The thermistor adopted in this work was a 100 kΩ NTC whose coefficients are $A=1.4276\times 10^{-3},\;B=2.3391\times 10^{-4},\;C=1.0649\times 10^{-7}$. Given the limited duration of all the experiments, namely shorter than 1 h, the temperature of the room did not change sensibly for the duration of the data acquisition, and hence the measured variation was induced by the heat transferred by the sensor.

Ambient light was measured with the OPT3004 ALS by Texas Instruments, which measures the ambient light in the range between 0.01 lx and 83 klx and features a very high rejection rate for infrared light, so its measurements are not affected by the light emitted by the ToFs. Given its 10 × 10 mm size, the ALS was mounted right above the VL53L5CX without occluding its measurements and to measure the incident light falling into its FoV. The ambient light was controlled with the office illumination and set to three different levels, i.e., full-brightness, dimmed, and turned off.

### 2.3. Experimental Uncertainty and Error Budget

To give context to the results and relative discussions presented in the following sections, we quantify the principal sources of uncertainty in the adopted experimental setup. The robotic manipulator exhibits a repeatability on the order of $10^{-1}$ mm. The target board position was measured using a tape measure and a protractor with nominal precisions of $10^{-1}$ mm and $0.1^{\circ }$, respectively. Additionally, the 3D-printed shell used to mount the sensors introduces geometric tolerances which we conservatively estimate to be within 0.3 mm (nozzle size of the adopted printer).

For the orientation measurements, the angular precision of $0.1^{\circ }$ corresponds to an angular uncertainty of $\delta \theta =0.1^{\circ }$, i.e., $1.745\times 10^{-3}$ rad. The induced error on the projected distance along the *x* axis is computed using the geometric relation

(2) $$\begin{array}{c} \Delta x_{\mathrm{ang}}=d\left(1-\cos(\delta \theta )\right). \end{array}$$

Considering the worst-case distance explored in this study, namely d=800 mm, the angular contribution can be up to $\Delta x_{\mathrm{ang}}\approx 0.0012\;\mathrm{mm}$, which is negligible compared to the translational positioning uncertainties.

Assuming independent contributions, the overall positioning uncertainty can therefore be approximated in quadrature as

(3) $$\begin{array}{c} \sigma_{\mathrm{tot}}=\sqrt{\sigma_{\mathrm{robot}}^{2}+\sigma_{\mathrm{manual}}^{2}+\sigma_{\mathrm{shell}}^{2}+\Delta x_{\mathrm{ang}}^{2}}\approx 0.33\;\mathrm{mm}, \end{array}$$

where $\sigma_{\mathrm{robot}}$ denotes the manipulator repeatability, $\sigma_{\mathrm{manual}}$ is the uncertainty associated with manual distance measurements, $\sigma_{\mathrm{shell}}$ is the geometric tolerance of the 3D-printed mounting structure, and $\Delta x_{\mathrm{ang}}$ is the projected positional error induced by angular uncertainty. The resulting value represents the resolution of the experimental setup. Accordingly, the sub-millimetre deviations reported in the following should be interpreted within this uncertainty bound.

### 2.4. Data Collection

To collect the data, we commanded the robot to occupy known positions with respect to the target board so to measure the true distance between this and the VL53L5CX being characterised. Once the sensor drift analysis was conducted, we waited for enough time in all the other experiments for the sensor measurements to settle. During each experiment, environmental conditions were not varied. The set of experiments was repeated for three different sensors to assess whether they displayed diverse behaviours. For all the analyses, the robot pose, VL53L5CX point clouds, thermistor, and ALS measurements were published on ROS nodes and collected in a synchronised fashion at 15 Hz. The following is a description of the executed tests.

- **Sensor Drift:** The robot was commanded at a distance of 250 mm with respect to the target, and 30 point clouds were acquired every minute for one hour. Data were collected starting from the moment the sensor was switched on.
- **Ambient light influence:** The robot was commanded in a known position and 100 point clouds were acquired in three different scenarios, i.e., full-brightness light, dimmed light, and with the light turned off. Since these analyses are meant to be leveraged mainly for indoor robotic manipulator tasks, we investigated whether office space illumination interferes with measurements. We also tested the sensor’s performance when a strong incandescent light source is emitted in the surroundings.
- **Within-frame distribution:** We assessed the deviation of the measured distances from the best-fitting plane interpolating them, averaging 30 point clouds acquired while the robot kept a known pose with respect to the target. This enabled measuring potential biases between the 64 beams, independent of distance or orientation.
- **Range influence:** We expanded the investigation proposed in [37], by monitoring the sensors’ temperature and the ambient light and by collecting samples with both a white and a black target board. The robot was commanded to move in steps of 5 mm for the 20–800 mm range to have more detailed results in this range with respect to what is available in the literature [6,36,37]. The height of the end-effector was kept constant at 400 mm from the base, and the orientation was not varied (as indicated by the red arrow in Figure 3). For the full range, the size of the target was such that the sensor measurements did not reach the surroundings, as the sensor pointed in the direction of the normal to the target. In each position, 100 point clouds were acquired. As mentioned in the Introduction, we investigated the 20–800 mm, as this is most sensible for robotic manipulators and obstacles in close proximity to mobile robots as well. Objects positioned at farther distances would fall out of the workspace, thus not interfering with task execution. For scene reconstruction purposes, instead, the error in the measurements and their sparsity would hinder task success for greater distances.
- **Orientation influence:** Similarly to the range analysis, we expanded on the preliminary characterisation proposed in [37], by considering targets of two different colours and by moving the robot at three different radial distances. By commanding the robot along a segment of a circle, the sensor was kept at a constant distance from the centre of the target, and just the mutual orientation varied. The segment was $40^{\circ }$ and spanned in intervals of $5^{\circ }$. The same experiment was conducted in the *x*-*y* and *x*-*z* planes (as highlighted in Figure 3), and the three distances were 150,200 and 300 mm. In the *x*-*y* plane analysis, the robot kept a 400 mm distance from the base. With these analyses, we investigated the combined influence of distance and orientation from the target. The explored ranges were chosen such that the sensor measurements fall on the target and not on the surroundings. In each position, 100 point clouds were acquired.
- **Material influence:** To assess whether material properties affect sensor measurements, we used different targets besides the white vinyl and the black sponge ones. We placed the sensors at three different distances, namely, 25, 40, and 60 cm, and collected 100 point clouds each time. In particular, measurements were taken when pointing at a cardboard attached to the board, a blue fabric cloth covering it, a wooden board without any wrapping or painting, a board with a grey laminate, and one covered with black vinyl. In all the tests, the target board was the only detectable obstacle.
- **Signal-to-noise ratio:** By exploiting the point clouds recorded for the within-frame distribution analysis, we retrieved an estimate of the signal-to-noise ratio, approximating it as the ratio between the mean measured distance for each pixel and the relative standard deviation. We further annotated the recurrence of rejected measurements in the diverse analyses.

## 3. Results

This section details the main findings obtained downstream to data collection. Following the experiments list introduced in Section 2, the results are collected in relative subsections.

### 3.1. Sensor Drift

**Figure 4:**
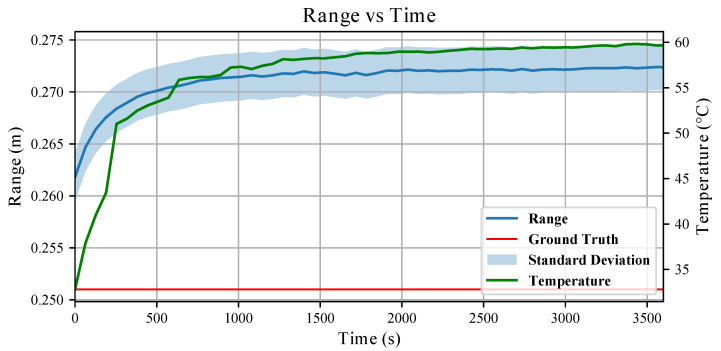
Sensor drift over 1h long experiment. The blue line represents the measured range, the red one denotes the ground truth distance between the target board and the VL53L5CX sensor, and the green one shows the measured temperature evolution on the surface of another VL53L5CX sensor throughout the test from the startup. Standard deviation on the mean measured range is shown with the shaded blue area.

In Figure 4, the evolution of the sensed range and of the sensor temperature over one hour is shown. Concerning the measured distance, 30 point clouds were collected every minute, $P_{i}^{j}$, with *i* defining the minute at which the point clouds were collected and j=1,…,30; for each point cloud, we computed the average of the 64 range measurements in the *x*-axis, $x_{i}^{jk}$, as in the following:

(4) $${\bar{x}}_{i}=\frac{1}{30}\frac{1}{64}\sum_{j=1}^{30}\sum_{k=1}^{64}x_{i}^{jk};$$

${\bar{x}}_{i}$ is plotted with the relative standard deviation in Figure 4.

It can be seen that the initial offset increases with time and settles after 900 s. Similarly, the standard deviation increases, meaning that when the sensor warms up, measurements becomes less repeatable. We also note that the temperature follows a similar pattern: at the beginning of the experiment, the sensor is at room temperature and has a steep increase in temperature in the first fifteen minutes. Even though the sensor temperature settles after almost half an hour, it is visible from the plot that measurements are less affected by the temperature increase when its derivative is closer to zero. Given the value of the average distance measured after one hour, ${\bar{x}}_{3600}$, we define $T_{99}$ the temperature at which the following is verified

(5) $${\bar{x}}_{T_{99}}=0.99{\bar{x}}_{3600}.$$

The tests conducted for the three sensors yield similar results, i.e., we get $T_{99}=51.61\;\pm \;1.70$ °C. It is important to note that, for the three tests, the starting temperature (the room temperature) has different initial values; thus, the time required for the VL53L5CX to reach $T_{99}∼52$ °C is slightly different. For all the other analyses, we waited t= 900 s before recording the measurements as the slowest time recorded to reach $T_{99}∼52$ °C is 890 s.

The bias observed in Figure 4, defined as the deviation of the average measured range from the ground truth, i.e., $\mathrm{bias}\left(t\right)=\bar{x}\left(t\right)-x^{*}$, exhibits a transient increase during the sensor warm-up phase. This temperature-induced drift can be approximated with a first-order exponential model of the form

(6) $$f\left(t\right)=A\times \left(1-e^{-t/T_{99}}\right),$$

where f(t) represents the time-dependent drift in mm component of the bias and *A* is the asymptotic bias variation induced during warm-up. The parameter *A* is identified as the difference between the steady-state bias and the initial bias measured at t=0 s. Accordingly, f(0)=0 mm and, for $t\gg T_{99}$, f(t)→A, meaning that the drift component of the bias saturates to a constant value.

**Figure 5:**
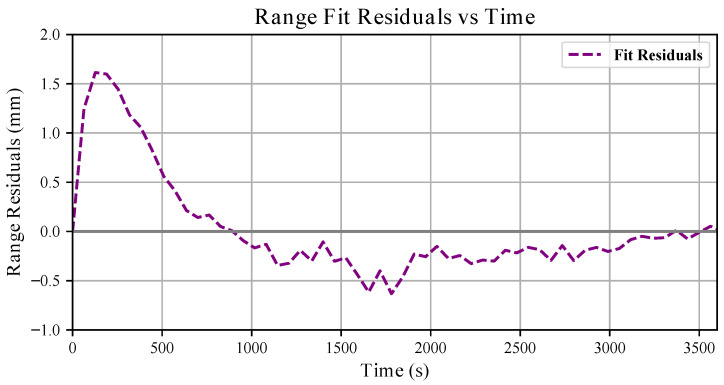
Range residuals over the 1 h long sensor drift experiment. The dashed purple line represents the difference between the measured bias degradation and the fitting exponential, f(t). The zero line is highlighted with a thicker grey line.

For the conducted analyses, we obtained A=0.01m. In Figure 5, we report the residuals between the measured bias and the fitted exponential model. The residuals remain consistently below 2mm and below 0.5mm for most of the experiment, indicating that the exponential model accurately captures the temperature-induced drift behaviour.

### 3.2. Ambient Light Influence

For this analysis, the robot was kept at a 200 mm distance from the white board, and 100 point clouds were acquired.

**Figure 6:**
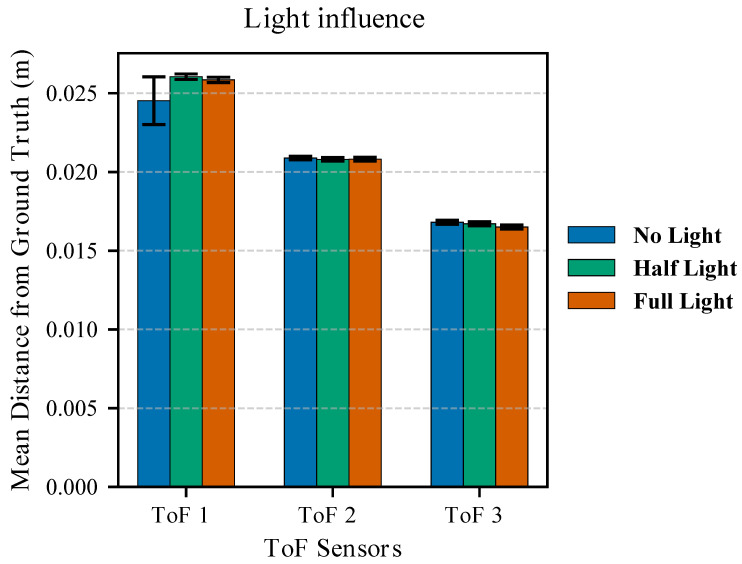
Lighting condition influence on depth estimation. For the three tested sensors, the mean distance of the measured depth from the ground truth is represented along with the standard deviation. Three lighting conditions are considered: office light off (no light), dimmed light (half light), and full office light (full light).

The measured incident light was ∼270 lux in full light conditions, between 100 and 50 lux when the office light was dimmed, and in a range between 10 and 0.5 lux when the office light was turned off. The test was repeated for three different sensors, and by placing the ALS sensor on top of the VL53L5CX used for data acquisition. The bar plot in Figure 6 shows how ambient light affects the VL53L5CX error on range estimation for the sensors. For this plot, range measurements were averaged, similarly to Equation (4), but with the due differences given that j=1,…,100 for this analysis; the standard deviation of the mean distance, ${\bar{x}}_{i}$,associated with each point cloud was computed and is shown in the figure with deviation bars. It can be seen that even if the incident light drops from 270 to 0.5 lx, the error on the estimation and the associated variability remain consistent. This behaviour applies to all the sensors, even though the mean distance from the ground truth differs; the causes of this are investigated in Section 2.4. As a consequence, even though for the other tests the ambient light assumed different values within the tested range, its effect on the sensor’s reading accuracy was considered negligible.

We further assessed VL53L5CX’s performance when exposed to the light diffused by a halogen lamp (500 W, ∼3 klux) placed beside the sensing unit, out of its FoV. The lamp diffuses light in the near infrared as well, causing sensor measurements to become particularly unreliable as further documented in Section 3.7, with a standard deviation of almost 16 mm.

### 3.3. Within-Frame Distribution

**Figure 7:**
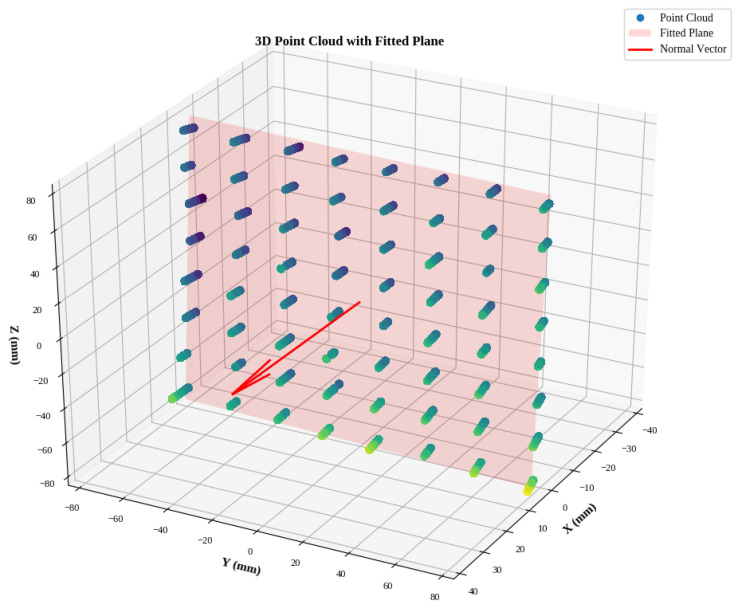
Deviation from best fitting plane. It is shown with the red shaded the best fitting plane for the depth measurements acquired with the sensor being at a fixed distance from the target board. Measured point clouds are displayed in the image with a lighter colour denoting further distance from the plane. The normal to the plane is shown with a red arrow.

Since the VL53L5CX yields depth estimation along equally distributed beams, we analysed within-frame variability in the measurements to assess if there are biases associated with the different beams. For this analysis, the robot was kept at a 200 mm distance from the white target board. In this case, we averaged the measurements along the beams and computed the best-fitting plane for the average distances using Singular Value Decomposition (SVD). In Figure 7, we plot the distribution of the measured points and the best-fitting plane to render the deviation of the measured distance of each beam, across 30 point clouds.

**Figure 8:**
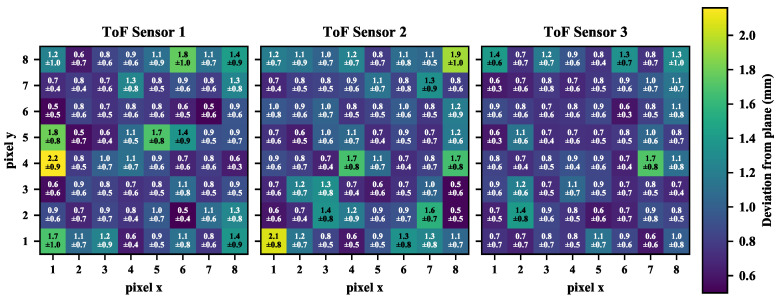
Within-frame deviation. The three matrices are associated with the three tested sensors and represent the distance from the best fitting plane. Each matrix cell details the mean distance and the relative standard deviation of each beam, which is identified as a pixel in an 8 × 8 image.

In Figure 8, we also quantify the mean and standard deviation of these discrepancies for the three tested sensors. It can be seen that the offset from the plane lies within a 0.5 to 2 mm range.

### 3.4. Range Influence

The range influence on measurement uncertainty was assessed for two different target materials: a white vinyl film and a black sponge. The experiments were repeated under identical conditions by changing only the side of the target board. As stated in Section 3.1, for all analyses the sensor temperature was above 52 °C, ensuring negligible thermal drift.

To estimate the effect of range on measurement uncertainty, the robot was commanded to move within the 20–800 mm interval. At each position, 100 point clouds $P_{i}^{r}$ were collected. For every point cloud, the average measured distance ${\bar{x}}^{r}$ was computed, and for each range *r* the mean and standard deviation across the 100 point clouds were evaluated.

**Figure 9:**
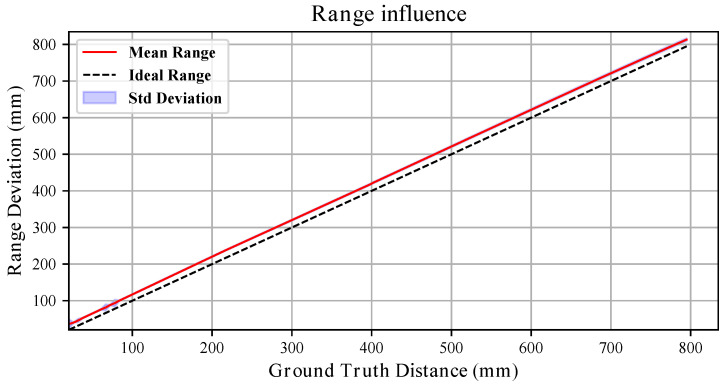
Range influence with the **white** board. The red line in the image interpolates the average depth measured at a given distance from the target board. The standard deviation across the 100 point clouds acquired at each pose is represented by the blue shaded area. The dashed line represents the bisector of the quadrant, which renders the ideal sensor measurements.

**Figure 10:**
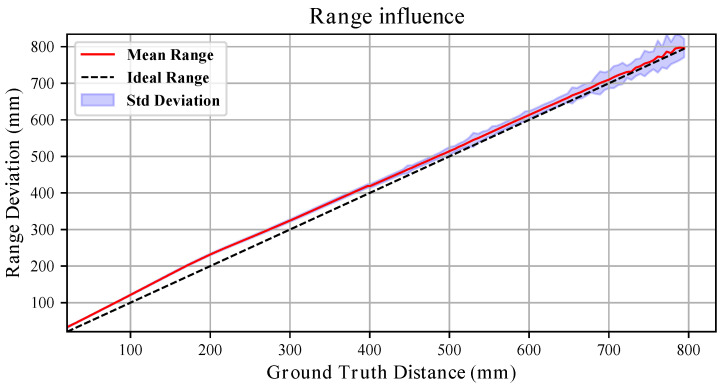
Range influence with the **black** board. The red line in the image interpolates the average depth measured at a given distance from the black target board. The standard deviation across the 100 point clouds acquired at each pose is represented by the blue shaded area. The dashed line represents the bisector of the quadrant, which renders the ideal sensor measurements.

Figure 9 and Figure 10 compares the sensor response for the white and black targets. In both cases, the dashed line represents the ideal sensor (bisector of the quadrant), while the red line interpolates the mean measured values.

For the white board, the response is well approximated by a first-order polynomial. For one representative sensor, we obtain

(7) $${\bar{x}}^{r}=1.005\;x^{*}+17.74\;\left(mm\right),$$

with $x^{*}$ being the ground truth. The deviation from ideal behaviour reaches a maximum of approximately 13 mm at 20 mm, then rapidly decreases and remains limited over most of the explored range. The overall linear trend is shared among the three tested sensors, with slopes {0.9939,1.0384} mm/mm and offsets {24.1,18.15} mm for the remaining units.

In contrast, when pointing at the black board, the trend deviates more from linearity. Interpolating the average range measurements with a first-order polynomial yields slopes {0.9766,0.9698,0.9763} mm/mm and offsets {27.79,32.08,32.42} mm. Compared to the white target, the slopes are systematically smaller and the offsets larger, indicating a material-dependent bias.

**Figure 11:**
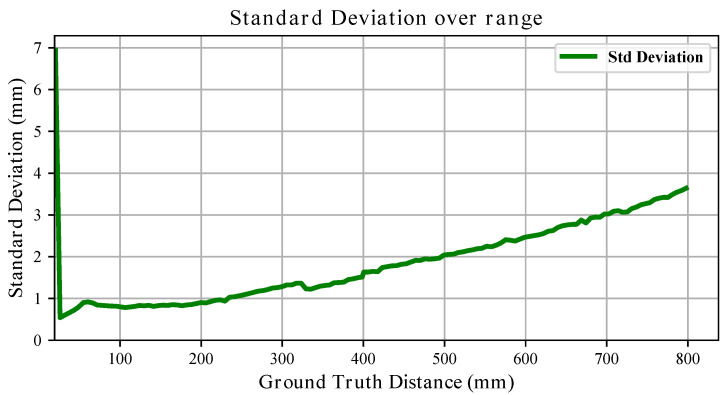
The evolution of the measured depth standard deviation over the full range when pointing at the **white** board is shown.

**Figure 12:**
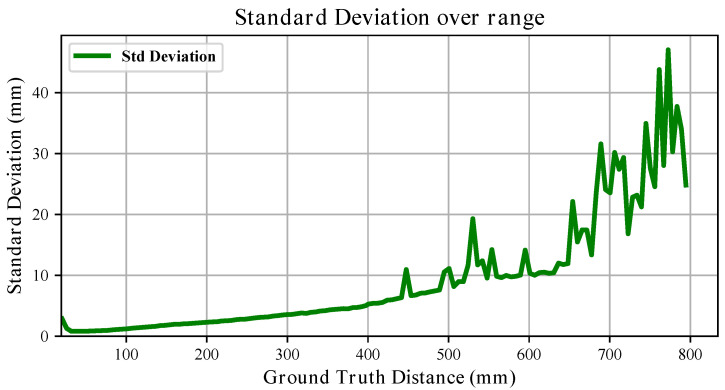
The evolution of the measured depth standard deviation over the full range when pointing at the **black** board is shown.

The standard deviation trends further highlight the influence of material. For the white board, the variability generally increases approximately linearly with distance (see Figure 11), remaining within a few millimetres across most of the range. For the black board, although the short- and mid-range behaviour resembles that of the white target, variability increases substantially beyond 400 mm, reaching peaks of nearly 50 mm (see Figure 12). Thus, while both materials exhibit range-dependent uncertainty growth, the degradation is markedly stronger for the black surface.

Overall, these results demonstrate that both systematic bias (slope and offset) and stochastic uncertainty are significantly influenced by the target material.

### 3.5. Orientation Influence

We extended the investigation proposed in [37] on the effect of mutual orientation between the sensor and the target. The analysis was conducted for both white and black targets, exploring a 40° range in the *x*-*y* and *x*-*z* planes (see Figure 3). Measurements were collected at three different radial distances. The sensor temperature was maintained above $T_{99}$, and range measurements were compensated for according to the linear models obtained in Section 3.4. At each commanded orientation *o*, 100 point clouds $P_{i}^{o}$ were collected. The deviation from the ground truth distance $x^{*}$ was evaluated along the robot *x*-axis.

**Figure 13:**
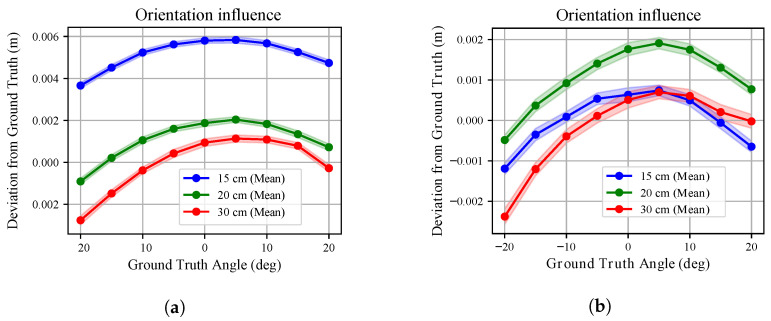
The orientation influence over depth measurements is shown for the *x*-*y* plane (**a**) and the *x*-*z* plane (**b**) analyses when pointing at the **white** target. The three colours identify the different radial distances from the target point at which the robot was kept during the analyses. The deviation of the average measured depth from the ground truth distance is shown with the relative standard deviation (shaded areas).

**Figure 14:**
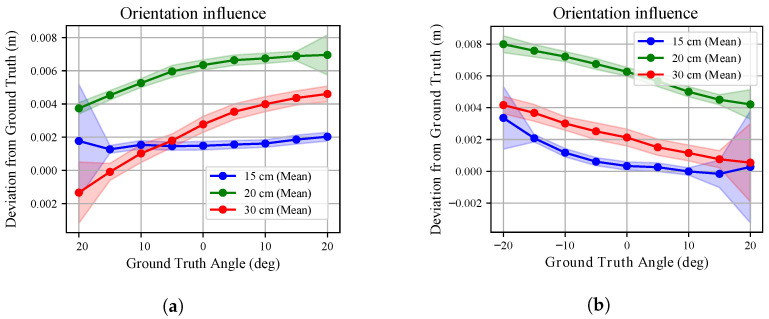
The orientation influence over depth measurements is shown for the *x*-*y* plane (**a**) and the *x*-*z* plane (**b**) analyses when pointing at the **black** target board. The three colours identify the different radial distances from the target point at which the robot was kept during the analyses. The deviation of the average measured depth from the ground truth distance is shown along with the relative standard deviation (shaded areas).

Figure 13 and Figure 14 compare the deviation from ground truth for white and black targets. For the white board (Figure 13), once compensated for range bias, the residual error remains limited across the explored angular range for all radial distances. For the black board (Figure 14), the degradation is more evident. The deviation from ground truth increases with orientation angle and is consistently larger than in the white-target case, confirming the material-dependent sensitivity already observed in the range experiments.

**Figure 15:**
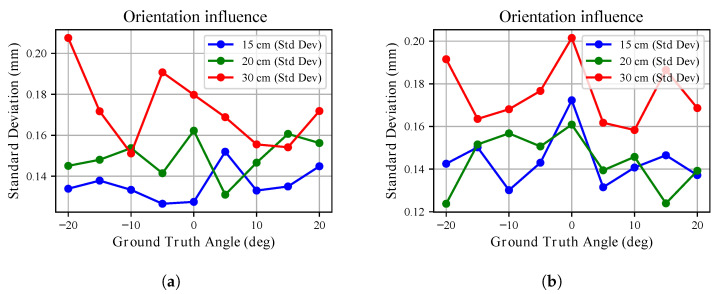
The orientation influence over depth measurements standard deviation is shown for the *x*-*y* plane (**a**) and the *x*-*z* plane (**b**) analyses when pointing at the **white** target. The three colours identify the different radial distances from the target point at which the robot was kept during the analyses. The standard deviation of the average measured depth is shown.

**Figure 16:**
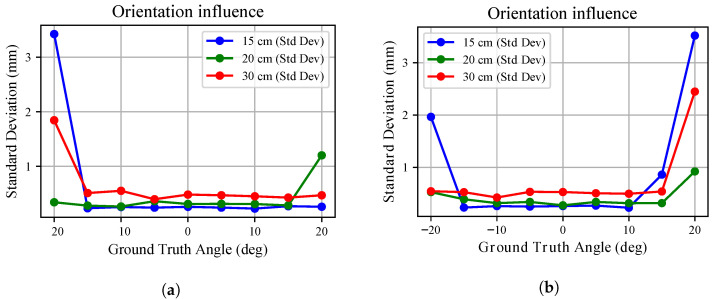
The orientation influence over depth measurements standard deviation is shown for the *x*-*y* plane (**a**) and the *x*-*z* plane (**b**) analyses when pointing at the **black** target board. The three colours identify the different radial distances from the target point at which the robot was kept during the analyses. The standard deviation of the average measured depth is shown.

The standard deviation trends in Figure 15 and Figure 16 further emphasize this behaviour. For the white board, the standard deviation remains below approximately 0.3 mm across all angles and distances. Similar behaviour is observed for the three tested sensors. However, these sub-millimetre deviations must be interpreted within the metrological bounds of the experimental setup as noted in Section 2. Consequently, no major influence on measurement uncertainty can be attributed to mutual orientation in this case. Conversely, for the black board, uncertainty is almost twice that observed for the white surface and increases more markedly at larger angles. Differences among the three sensors remain limited.

These findings confirm that even the mutual orientation effect is strongly influenced by surface properties: while geometric misalignment alone has a limited impact after range compensation, reduced reflectivity amplifies uncertainty.

### 3.6. Material Influence

**Figure 17:**
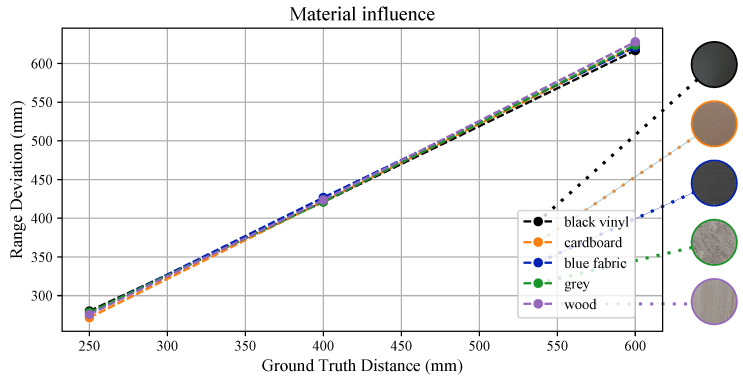
Material properties influence on range estimation. It is shown the estimated range at three different distances for five materials: black vinyl, cardboard, fabric, grey laminate, and wood. The right-hand side further details the adopted material appearances.

Besides the results presented in Section 3.4, where the sensors pointed at a white vinyl and black sponge targets, we also investigated how different materials affect range measurements. Figure 17 highlights the average estimated range, and it can be seen that the linear trend observed previously is displayed even with different materials. In Table 1, instead, we report the standard deviations exhibited for different materials. These results deviates from what obtained with the white board target within setup’s uncertainty bounds in all the cases except for the black vinyl board. In this case, as detailed further in Section 3.7, uncertainty was almost double on average, and the sensor’s performance degraded heavily, as most results were flagged as unreliable.

### 3.7. Signal-to-Noise Ratio

For every depth measurement, the VL53L5CX sensor yields a range statusflag stating whether the estimation should be considered or discarded as noise. Throughout the entire data collection, we collected these flags and listed them in Table 2 by grouping experiments aimed at assessing the influence of a certain factor for each VL53L5CX. It can be seen that the percentage of rejected measurements is similar for the three sensors across the different experiments. The highest rejection rate was obtained during tests that were aimed at analysing the influence of mutual orientation between the target and the sensor.

Among the tested conditions, two experiments produced very different results and were left out of the table to avoid interference with the common trends experienced across the tests. Precisely, unreliable measurements were obtained when the sensor (i) was exposed to the intense light of the halogen lamp and (ii) pointing at the black vinyl target. In the first case, the measurement rejection rate was almost 45%. When pointing at the black vinyl target, instead, just 18.8% of the measurements were flagged as reliable at a 25 cm distance, 20.3% at 40 cm, and 34.4% at 60 cm.

**Figure 18:**
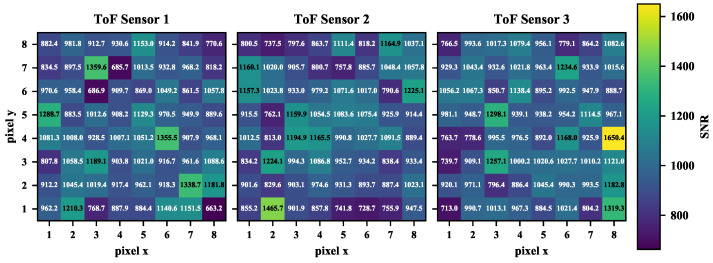
Estimation of the signal-to-noise ratio for the three tested VL53L5CX sensors. The average signal-to-noise ratio is computed along each beam, considering 30 point clouds. Beams are identified as cells of an 8 × 8 matrix. The colour bar shows that greater signal-to-noise ratio values are associated with a lighter colour.

We also computed an approximation of the signal-to-noise ratio which is not provided in the datasheet. To this end, we considered 30 point clouds acquired with the robot keeping a 200 mm distance from the white target board. For each measurement along the beams, we computed the average measured distance and divided it by the standard deviation across the 30 measurements. In Figure 18, this distribution is shown for the three tested sensors with 8 × 8 matrices. The signal-to-noise ratio varies within a ∼800–1650 range, thus confirming the sub-millimetre measurement repeatability observed for this range in Section 3.4 with a white board. No noticeable pattern is shared across the sensors and among the different beams.

## 4. Discussion

With what is presented in Section 3, we intend to complement the information given in the datasheet of the VL53L5CX with a robust characterisation, especially in the short and medium range, namely below 1 m. We investigated this range, as detailed in Section 1, because of the attention these multizone proximity sensors have received for robotics applications. In the following we discuss main findings, limitations, and future work.

### 4.1. Key Findings and Practical Implications

Notably, with our analyses, we found that the VL53L5CX sensor embedded in its off-the-shelf PCB tends to warm up quickly, reaching temperatures above 50 °C in a few minutes. This severely affects measurement accuracy since the standard deviation increases with the rising of the temperature and the measured depth drifts from the ground truth. Such behaviour may be induced by the adoption of the continuous data stream mode adopted throughout the experiments, which allows to constantly stream new data at 15 Hz. In addition to this, the current VL53L5CX 3D-printed housing allows for enough room around the sensing unit, but future designs will leave more room to avoid the overheating of the whole PCB.

In Section 3.1, the proposed exponential model provides a parametric description of the temperature-induced drift from the start-up. The residual analysis (see Figure 5) shows that the discrepancy between the measured bias and the fitted curve remains below 2mm and below 0.5mm for the majority of the experiment. As a result, measurements can be compensated at the start-up using this fitted model, and the residual bias will depend mainly on the range distance from the target. Otherwise, waiting for a warm-up period before collecting data is sufficient considering that the VL53L5CX sensors produced consistent measurements after a few minutes (see Section 2.4).

Section 3.2 highlighted how normal office light conditions do not have a major effect on sensor measurements, while intense incandescent light sources can hinder depth estimation sensibly. This is to be expected since, in such cases, light is emitted also in the infrared zone, thus interfering with the beams emitted by the sensor. It is then relevant to avoid sensor exposure to similar light sources to avoid measurement rejection rates as high as 45%.

In Section 3.3, we observed no substantial differences across the different beams, as they spread around the best fitting plane within a 2.5 mm distance without any noticeable repeating pattern across beams and among sensors. Because of this, it can be assumed that there is no particular per-beam bias but rather a similar behaviour in depth estimation along each beam.

For what concerns depth estimation, in Section 3.4 we showed that sensors produce depth measurements with an offset bias with respect to the ground truth, which is contained within 3 cm, and an almost perfectly linear deviation from what an ideal sensor would produce. With respect to what is presented in the datasheet, the measured standard deviation was mostly contained within 1% of the measured range when the sensors were pointing at the white board. When facing the black board, instead, measurements repeatability showed a different trend, especially in the medium range with peaks of ∼6%, which is still below what is reported in [24], i.e., 11% when pointing at a grey target. These findings allow for compensation of the measurements and become important when modelling these sensors, as already proved in [37]. However, if high precision is needed, the variability in the parameters defining sensor performance identified in Section 2.4 cannot be overlooked and per-sensor characterisation is paramount. Further, the diversity in results obtained when using either a white or a black board suggests that different materials and colours yield very different performance. Therefore, an uncertainty model for an arbitrary material is insufficient and broader investigation on material reflectivity effects can be necessary depending on the applications the VL53L5CX is adopted for.

Conversely, we noted that mutual orientation between target and sensor does not influences heavily depth measurements. The point clouds, once compensated for according to what was found Section 3.4, showed little deviation from ground truth, consistently below 1 cm and with a standard deviation often smaller than 1 mm. Larger deviations were recorded when pointing to the black board with angles above 15 deg. Accordingly, the mutual orientation effect on measurements can be ignored if sub-millimetre precision is not sought.

In Section 3.6, we noted how material properties do not affect sensor measurement sensibly in most cases; thus, range-dependent sensor’s uncertainty can be safely modelled following the results of Section 3.4, rather than a material-dependent bias. However, it must be noted that when pointing at darker objects, sensor performance degrades as experienced in the case of the black sponge and vinyl targets. In this last case specifically, most measurements were flagged as not reliable in the close-proximity range, which is detrimental when the sensor is adopted for safety tasks such as obstacle avoidance. This suggests that, in similar cases, the presence of objects with similar properties should be avoided in the robot’s surroundings. Besides these edge cases, from Table 2, it can be noted that during the most extended tests, those conducted to assess how range and orientation affect measurements, the highest percentage of invalid measurements was obtained, namely up to almost 8%. The rejected data were evenly spread across these tests. Since these experiments reproduce a more realistic applicative scenario—one in which the distance from the target constantly changes—it can be assumed that for extended experiments a near 10% rejection rate can be achieved. This is a relevant aspect to replicate when modelling and simulating sensor performance, as some measurements can be randomly rejected during data collection if the range status flag is considered.

### 4.2. Limitations and Avenues for Further Work

In this study, we did not consider the effect that highly reflective materials have on the measurements, and we did not investigate noise induced by multiple path reflections. These effects were observed for the VL53L5CX sensors in [22], where reflective materials (aluminium bars) and corners induced artifacts in the measured point clouds, and analysed for other time-of-flight cameras in [41,42].

Moreover, despite the lighting conditions were varied in a wide range in our data collection, outdoor scenarios can pose challenges to the VL53L5CX. For instance, infrared light coming directly from the sun can interfere with the working mechanism of the sensor, similarly to the light source adopted in Section 3.2, and is expected to hinder reliable depth estimation. This aspect cannot be ignored if the sensors are deployed outside of laboratories or controlled environments, e.g., for agricultural robotics tasks.

Finally, we note that we tested just three sensors and the adopted setup does not allow sub-millimetric precision in the estimation of the ground truth as described in Section 2.3. Accordingly, in our analyses, we did not compensate for residual errors that could have been induced by tolerances in the placement of the sensors, as well as target flatness. Therefore, a higher-precision metrology, such as optical distance references or calibrated positioning rigs, is needed to decouple sensor performance from setup-induced uncertainty. Nevertheless, so far, these sensors have not been deployed for tasks in which sub-millimetric precision is necessary.

Future analyses will be aimed at characterising multiple paths induced noise when pointing at corners, and assessing the effect of direct exposure to infrared light in outdoor environments.

## 5. Conclusions

In this work, we presented a systematic characterisation of the miniaturised ToF sensor VL53L5CX, targeted for indoor robotics applications. We mounted three different sensors on a robotic manipulator to precisely control the pose of the sensor with respect to a target board and conducted experiments on sensor drift, ambient light influence, within-frame beam variability, range accuracy, and orientation sensitivity. We also computed an empirical signal-to-noise ratio and documented the occurrence of invalid measurements throughout the entire data collection. The point cloud data structure was adopted to process depth measurements in all the experiments, and both white and black target boards were considered. We identified key findings that are not fully documented in the manufacturer’s datasheet. In particular, it was noted that the VL53L5CX undergoes a warm-up transient that lasts almost 15 minutes and the depth measurements present a near-linear range bias. Degraded performance was noted when the sensors were pointing at targets covered with black sponge and vinyl, and when incandescent light sources were emitting in the proximity of the sensor. Conversely, varying the office lighting conditions did not raise notable effects on the estimation of depth. We further observed that within-frame beam variability in the measurement is negligible. Based on these observations, it is possible to improve depth measurement by adopting range-induced error compensation, waiting a warm-up time before collecting data, and assessing if the reflectivity of the surrounding objects visibly affects measurements. Future work will expand characterisation to multipath and highly reflective surfaces, and evaluate outdoor illumination conditions to support wider deployment in both manipulation and mobile robotics.

## Figures and Tables

**Table 1:** Standard deviation of range estimation (in mm) for each material at three different distances.

Material	σ mm		
	**25 cm**	**40 cm**	**60 cm**
Black Vinyl	5.158	5.839	11.161
Cardboard	3.206	5.110	6.453
Blue Fabric	2.378	4.049	5.732
Grey Laminate	2.800	4.945	7.131
Wood	2.856	4.623	5.810

**Table 2:** Range status counts grouped by experiment cluster and VL53L5CX sensors. “Keep” denotes a valid depth measurement, while “Reject” denotes an unreliable measurement. “Valid (%)” values represent the occurrence rate of valid measurements in the experiments.

ToF	Experiment Clusters	Keep	Reject	Valid (%)
1	Light influence (full/half/no)	28,736	64	99.78
	Range	2,797,256	187,003	93.75
	Orientation (*x*-*y* & *x*-*z*)	974,131	61,375	94.08
	Sensor drift	110,656	576	99.48
2	Light influence (full/half/no)	28,672	128	99.56
	Range	2,787,519	147,916	94.97
	Orientation (*x*-*y* & *x*-*z*)	956,541	80,154	92.27
	Sensor drift	110,976	384	99.66
3	Light influence (full/half/no)	28,800	0	100.00
	Range	2,851,421	75,825	97.41
	Orientation (*x*-*y* & *x*-*z*)	983,024	53,635	94.82
	Sensor drift	110,976	256	99.77

## Data Availability

The original contributions presented in this study are included in the article. Further inquiries can be directed to the corresponding author.

## Abbreviations

The following abbreviations are used in this manuscript:

ToF	Time-of-Flight
SPAD	Single Photon Avalanche Diode
FoV	Field-of-View
SLAM	Simultaneous Localisation and Mapping
ALS	Ambient Light Sensor
NTC	Negative Temperature Coefficient
HRI	Human–Robot Interaction
SVD	Singular Value Decomposition
MPI	Multi-Path Interference
PCB	Printed Circuit Board
